# Vitamin D deficiency in young children with iron deficiency in Misan province, Iraq

**DOI:** 10.25122/jml-2021-0264

**Published:** 2022-03

**Authors:** Salah Hashim AL-Zuhairy, Mohammed Abed Darweesh, Mohammed Abdul-Mounther Othman, Noor AL-Huda Salah AL-Zuhairy

**Affiliations:** 1. Department of Pediatrics, College of Medicine, University of Misan, Amarah, Iraq; 2. Department of Biochemistry, College of Medicine, University of Misan, Amarah, Iraq; 3. Department of Pharmacy, Al-Manara College for Medical Sciences, Amara, Iraq

**Keywords:** ferritin, vitamin D, anemia, toddler.

## Abstract

This study aimed to assess vitamin D status and its association with iron status in young Iraqi children. A total of 95 infants and toddlers with iron deficiency (ages ranging from 6 to 24 months) and an equal number of 95 healthy subjects with normal hemoglobin (Hb) and sufficient ferritin level with matching age were included as a control group. A specially designed questionnaire was used to collect data. The cases were classified into iron deficiency (ID) and iron deficiency anemia (IDA) according to hemoglobin and ferritin levels. The cases and control groups were subdivided into vitamin insufficiency (VDI), vitamin D deficiency (VDD), and vitamin D sufficiency groups according to 25-hydroxyvitamin D [25(OH)D] levels. Young children with IDA have significantly lower serum levels of 25(OH) D compared with ID and control groups (p<0.05). According to iron status, VDI and VDD were present in 20% and 70% of IDA, 25.7% and 60%of ID, and 26.3% and 30.5% of control groups, respectively, with a significant difference in vitamin D level (p<0.05) among studied groups. A significant positive correlation (p=0.000) was found between serum ferritin level and 25(OH) D level in studied patients. Young children with severe iron deficiency have a higher prevalence of vitamin D deficiency, and there was a significant positive correlation between serum ferritin level and 25(OH) D levels among studied children.

## INTRODUCTION

Iron and vitamin D deficiencies are the two most common nutritional deficiencies in children in developing countries. However, these deficiencies are frequently overlooked and can significantly affect a child's overall health, impacting the health of communities [1–2]. Data from World Health Organization (WHO) showed that anemia prevalence among children aged 6–59 months was 42.6% globally in 2011,48.6% in the Eastern Mediterranean Region, and 36% in Iraq [3] whereas in the US and UK, 30% of children aged 1–2 years had iron deficiency (ID) and 10% had iron deficiency anemia (IDA) [3–5]. There is insufficient data on the epidemiology and burden of iron deficiency, but Rasheed [6] reported that 52% and 48% of Iraqi children aged 1–5 years from the northern region suffer from iron deficiency according to iron saturation in the blood and transferring, and IDA was severe in the younger age group (12–24 months). The outcomes of iron deficiency are many and serious, affecting children's health and the development of societies and countries. IDA in early childhood is associated with impaired neurocognitive function and exercise intolerance, and the sequels of iron deficiency may continue even after its successful treatment [6]. Vitamin D is a pro-hormone made in the skin and then activated in the liver and kidneys and is for bone mineralization. Vitamin D deficiency can lead to rickets in children, mainly occurring at age 3–18 months [7]. The high prevalence of low serum vitamin D levels is a global concern among children even in areas with good sun exposure, and vitamin D deficiency (VDD) and insufficiency (VDI) is common among otherwise healthy young children. Gordon *et al.* [8] found that the prevalence of vitamin D deficiency was 12.1% among infants and toddlers, and 40.0% had insufficient levels.

Iron deficiency anemia (IDA) and vitamin D deficiency (VDD) have been observed simultaneously in infancy and young children [9–11]. The relationship between these two deficiencies is unclear due to their linked metabolisms, and it is not easy to assess which of these nutrients has the most substantial effect on the other but appears to be reciprocal [12–17]. Iron participates in the second hydroxylation step of vitamin D activation. The conversion is done by a renal 25-hydroxyvitamin D 1α-hydroxylase (1α-OHase) enzyme, containing a cytochrome P-450 (CYP2R1 and CYP27B1 respectively), a ferredoxin, and a ferredoxin reductase. Consequently, less available iron could affect the production of the active form of vitamin D [18].

On the other hand, vitamin D modulates the risk of anemia by decreasing inflammation and stimulating Erythropoiesis, as 1,25(OH)2D plays a role in RBCs synthesis by its effect on the erythroid progenitors [18–20]. There is a metabolic association between iron status and 25-hydroxyvitamin D levels through fibroblast growth factor 23 (FGF23), which affects 1α-OHase enzyme expression, and its levels increase with low serum 25-hydroxyvitamin D [21–22]. However, there is no data about the prevalence of vitamin D deficiency among young children in Iraq. In the current study, we would like to explore the association between biomarkers of iron with vitamin D levels in patients with iron deficiency states. Determination of serum ferritin and 25(OH) D levels were compared between ID, IDA, and healthy control children, and the data were analyzed to shed light on the correlations between these micronutrients deficiencies [23–24]. We hypothesized that the results could draw more attention to pediatricians and health authorities to seek these combinations of deficiencies and prevent and treat this emerging health problem.

## MATERIAL AND METHODS

The study was conducted from 1^st^ July to 31^st^ December 2019, a time with a substantial amount of sunlight in Iraq. A total of 190 infants and toddlers between the age of 6 and 24 months seen at the outpatient clinic of Maternity and Child Welfare Hospital-Misan were included in the study. This hospital is the primary public hospital in the Amarah, Misan Province, located about 336 km south of Baghdad, Iraq. The study sample enrolled 95 cases that were sub-classified to 35 children with iron deficiency (ID) and 60 patients with iron deficiency anemia (IDA), and an equal number of 95 healthy young children with normal hemoglobin (Hb) and sufficient ferritin level with matching age and sex, who attended the child visit or health checkups were included as a control group. In order to rule out conditions that may have a potential effect on iron status biomarker (ferritin), young children with sick febrile illness (increased CRP), seriously ill cases, malnutrition, chronic diseases, Hemoglobinopathies, and children receiving supplements were excluded from the study. Height and weight were measured, and body mass index (BMI) was calculated using the formula (BMI=kg/m^2^), where kg is the weight in kilograms of the infant or the toddler and m^2^ is their length in the square meter. Venous blood was drawn by venipuncture, 2 milliliters in potassium EDTA tube for complete blood count (CBC) using ABXMicros ES 60 hematology analyzer ((Horiba Medical, Montpellier, France). Another 3 milliliters of venous blood were for serum separation after centrifugation at 1000 g for 15 minutes and stored at -20℃. Serum ferritin was measured by an enzyme linked fluorescent assay (ELFA) method using VIDAS IVA 30/908, and ferritin kit, (BioMérieux, Marcy l'Étoile, France). Combination of an enzyme immunoassay sandwich method with a final fluorescent detection Enzyme-Linked Fluorescent Assay (ELFA) using 25-Hydroxy Vitamin D Kit (BioMérieux, Marcy l'Étoile, France) was used for serum vitamin D measurement. All assays were done according to the manufacturer's protocol and performed in duplicate. IDA was defined as Hb ≤11 g/dl, mean corpuscular volume (MCV) (≤70 fL), RDW elevation ≥15%, and ferritin ≤12 ng/mL. ID was defined as Hb>11 g/dL and ferritin ≤12 ng/mL [24]. VDD was defined as 25(OH)D ≤20 ng/mL, VDI as 25(OH)D of 20–30 ng/ml, and VDS as 25(OH)D >30 ng/mL [25]. Statistical analyses were reported as mean estimation±standard deviation (SD), numbers, and percentage (%). Statistical Package for Social Sciences (SPSS) version 23 for windows was used for analysis. Comparison of categorical data was carried out by Chi-square test and one-way analysis of variance (ANOVA). A p-value of <0.05 was considered statistically significant.

## RESULTS

Over a 6-month period, 35 and 60 young children met the criteria of ID and IDA, respectively, and 95 healthy children as control group were involved in this study. The mean age of children in the ID group (10.1±6.4 months) was lower than the mean age in the IDA (11±5.88 months) and control (11.3±5.95 months) group, with no significant differences in age of subjects (p=0.8). There were no significant differences among the 3 groups regarding subjects' height and body mass index (p=0.3). However, the weight of infants and toddlers was significantly different among groups, with the IDA group having a lower mean weight than the ID and the control group (Table 1).

**Table 1.** General characteristics of ID, IDA, and control groups.

Variables	ID Patients (n=35)	IDA (n=60)	Controls (n=95)	p-value
**Age (mo.)**	10.1±6.4	11±5.88	11.3±5.95	0.8
**Length (cm)**	68.8±10.05	64±8.64	70±10.25	0.3
**Weight (kg)**	7.5±2.55	7.2±1.97	8.8±2.65	0.04
**BMI (kg/m^2^)**	16.8±7.67	18.8±7.83	19.2±9.02	0.3

n – numbers; ID – iron deficiency; IDA – iron deficiency anemia; mo – months; cm – centimeters; kg – kilogram; BMI – body mass index; m^2^ – meter square. Value is presented as Mean±SD; p values were obtained using one-way analysis of variance (ANOVA) test.

Regarding the hematological and biochemical {ferritin and 25(OH) D} profiles in studied groups, the mean RBC count, Hb, PCV, MCV, RDW, Retic %, ferritin level, and 25(OH) D level were significantly lower in ID and IDA groups (p<0.05) as compared to control group, but platelets count in the ID and IDA patients were significantly higher (p<0.05) compared to the control group (Table 2).

**Table 2.** Differences in hematological and biochemical profiles among ID, IDA, and control groups.

Variables	ID Patients (n=35)	IDA (n=60)	Controls (n=95)	p-value
**Red blood cells (million/mm^3^)**	4.4±0.7	3.7±0.3	5.2±0.4	0.000
**Hemoglobin (g/dl)**	11.1±0.8	8.7±0.9	12.3±1.2	0.005
**PCV %**	33±2.1	25±2.2	36±2.9	0.01
**MCV (fl)**	69.2±5.1	66.4±3.7	78.6±2.8	0.000
**RDW %**	13.8±2.1	19.1±2.1	13.3±1.4	0.0005
**Reticulocytes %**	0.6±0.02	0.4±0.08	0.9±0.01	0.000
**Platelets (10^9^/L)**	331.5±139.4	406.7±211.8	248.6±193.6	0.03
**Ferritin (ng/ml)**	9.8±3.5	8.6±2.0	38.3±29.8	0.000
**25(OH) D (ng/ml)**	14.8±4.5	11.5±2.7	34.1±5.5	0.000

n – numbers; ID – iron deficiency; IDA – iron deficiency anemia; Hb – hemoglobin; PCV – packed cell volume; MCV – mean corpuscular volume; RDW – red cell distribution width; 25(OH) D – 25 hydroxy-vitamin D. Value is presented as Mean±SD; p values were obtained using one-way analysis of variance (ANOVA) test.

**Figure 1.**
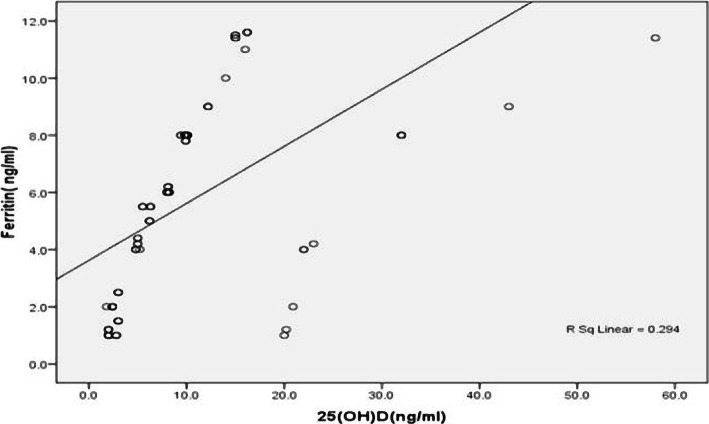
Pearson's correlation between serum ferritin levels with 25(OH) D levels in iron deficiencies state.

The 25(OH)D levels were estimated in all subjects, and vitamin D deficiency was observed in 48.4% (92) of subjects, VDI was seen in 24.2% (46) children, while 27.4% (52) subjects had normal vitamin D levels. Analysis of vitamin D status for infants and toddlers in different study groups revealed that in the IDA group, VDD and VDI were seen in 70%, (42/60) and 20% (12/60) respectively; in the ID group, VDD and VDI were observed in 60%(21/35) and 25.7% (9/35) respectively, while in the control group VDD and VDI were seen in 30.5% (29/95) and 26.3% (25/95) respectively. There was a statically significant difference in the levels of vitamin D {25(OH)D} among infants and toddlers in different studied groups (p=0.0002) (Table 3). There was a significant positive correlation (r=0.542, p=0.000) between serum ferritin and 25(OH) D (Figure 1).

**Table 3.** Differences in vitamin D status among ID, IDA, and control groups according to their 25-hydroxy vitamin D levels.

Vitamin D status	ID (n=35)		IDA (n=60)		Controls (n=95)		Total		p-value
	N	%	N	%	N	%	N	%	
**VDD**	21	60.0	42	70	29	30.5	92	48.4	0.0002
**VDI**	9	25.7	12	20	25	26.3	46	24.2	
**VDS**	5	14.3	6	10	41	43.2	52	27.4	

n – number; ID – iron deficiency; IDA – iron deficiency anemia; VDD – vitamin D deficiency; VDI – vitamin D insufficiency; VDS – vitamin D sufficiency. Value is presented as Mean±SD; p values were obtained using Chi-square test.

## DISCUSSION

This is the first study in Iraq to assess the prevalence of vitamin D deficiency in infants and toddlers with iron deficiency and evaluate any coexisting correlation between a biomarker of iron status (ferritin) and vitamin D level. Iron and vitamin D are two essential micronutrients for the normal growth of young children, and their deficiencies are still a major health problem in developing countries, including Iraq. Moreover, these deficiencies can significantly impact the physical development, immunity, increased risk of infection, and neurocognitive development of children, and these unwanted consequences persist even in asymptomatic cases and after successful treatment [1–5]. Our study agrees with other studies that observed a significantly high prevalence and coexisting correlation between ID and IDA with VID and VDD [1, 9, 10, 12, 13]. Our finding can be attributed to decreased vitamin D intestinal absorption caused by iron deficiency, as Heldenberg *et al.* [14] observed that VDI and VDD were seen in more than 50% of infants with IDA although they received daily supplementation with vitamin D. In addition, they mentioned that severe IDA might cause malabsorption syndrome, and mild IDA may impair fat absorption, including vitamin D, which is a fat-soluble vitamin and hence decrease vitamin D levels in plasma. The positive relationship between these two elements is advanced by the increased vitamin D concentration in infants after intramuscular iron replacement. A metabolic association between iron status and 25-hydroxyvitamin D levels through fibroblast growth factor 23 (FGF23) was supported by recent studies [21, 22]. In addition, Katsumata *et al.* [18] noticed that severe IDA could influence bone deposition and resorption by deactivating the synthesis and metabolism of iron-dependent enzymes, resulting in decreased plasma 1,25(OH)2 D levels. Alternately, other researchers assessed iron status according to vitamin D levels in children and observed a positive coexisting association between these two micronutrients [16–19]. Hence, it appears that the metabolism of vitamin D is dependent on iron, although it is not well known how severe IDA is and at what serum ferritin level the risk of developing VDD begins to increase significantly.

So, vitamin D status evaluation is needed for pediatric patients with ID and vice versa. Unlike the current study findings, Albar *et al.* [15], Yoon *et al.* [17] and Abdul-Razzak *et al.* [23] could not observe a significant correlation between anemia and VDD deficiency in their studies. Well-known risk factors can affect infants and toddlers' iron and vitamin D status, including age, weight, and BMI. In the current study, concerning potential determinants of ID and IDA, age, height, and BMI of infants and toddlers did not appear to significantly differ, while weight had a significant difference among studied groups [24, 25]. In comparison to our results, Jin *et al.* [9] reported similar findings regarding age, but BMI values in their study were higher and significantly related to iron status among infants aged 3–24 months. Unlike our results, Rasheed [6] found that the weight did not significantly affect iron status. The large body size of infants and toddlers does not necessarily indicate that the subject is not anemic, as faster growth without an iron supply might result in anemia. In addition, low body weight most likely reflects a state of malnutrition among our studied subjects, and protein deficiency can lead to IDA and VDD by decreasing the concentration of vitamin D and iron-binding protein in blood [9]. Malnutrition that leads to vitamin D disorders may sometimes be due to pathological states, such as celiac disease, exacerbated by vitamin D deficiency when children are on a gluten-free diet [26]. There are limitations in the current study, as it does not represent all pediatric populations.

## CONCLUSION

In conclusion, our study demonstrated that young children with iron deficiency have a higher prevalence of vitamin D deficiency, and there was a significant positive correlation between ferritin as an iron biomarker and vitamin D status among studied children with ID and IDA. Primary care physicians and pediatricians should be aware of VDI and VDD in children with iron deficiency and measure vitamin D levels, which is not currently a part of routine child healthcare, and supplement vitamin D and iron when necessary. Further studies including new markers of iron metabolism (such as hepcidin, soluble transferrin receptor) are recommended.

## ACKNOWLEDGMENTS

### Conflict of interest

The authors declare no conflicts of interest.

### Ethics approval

The study protocol was approved by the Ethical Committee at the College of Medicine, University of Misan, Iraq (162, 4^th^ June 2019).

### Consent to participate

Informed consent was obtained from parents or guardians of infants or toddlers included in the study.

### Personal thanks

The author is thankful to Dr. Hmood M. Hassan (Department of Public Health, Misan Health Directorate, Amarah, and Misan, Iraq) for his assistance in performing the statistical analysis.

### Authorship

SHA-Z is the corresponding author and was in charge of data collection, manuscript conceptualization, writing, data analysis, and manuscript submission and revision. MAD and NASA-Z contributed to data collection. MAMO contributed to data collection and data analysis.
